# Lack of Neuropsychological Deficits in Generalized Social Phobia

**DOI:** 10.1371/journal.pone.0042675

**Published:** 2012-08-03

**Authors:** Scott R. Sutterby, Jeffrey S. Bedwell

**Affiliations:** Department of Psychology, University of Central Florida, Orlando, Florida, United States of America; University of Granada, Spain

## Abstract

There are relatively few existing studies examining neuropsychological functioning in social phobia (SP), which collectively yield mixed results. Interpretation of results is further complicated by a number of methodological inconsistencies across studies, including the examination of neuropsychological domains in relative isolation from one another. The present study utilized a broader collection of neuropsychological tests to assess nine domains of functioning in 25 individuals diagnosed with generalized SP and 25 nonpsychiatric controls (NC). A mixed ANOVA revealed neither a significant group by domain interaction, nor a significant main effect of group. Furthermore, no significant group differences emerged between the SP and NC groups within each specific neuropsychological domain. These findings suggest that underlying neuropsychological deficits are not likely to account for the information processing biases observed in the empirical literature, and appear to be consistent with current theoretical models which argue for the specificity of these biases to social information.

## Introduction

Current theoretical models of social phobia (SP) emphasize specific cognitive biases in attention, interpretation, and memory which emerge during the processing of socially-relevant information and function in a cyclical manner to develop and maintain SP [1], [2]. Indeed, there has been a great deal of empirical support for attention biases [3]–[9] and interpretation biases [10]–[17], as well as preliminary evidence for specific memory biases [18]–[21], for socially-relevant information among SP patients as compared to nonpsychiatric controls. These cognitive models could be further strengthened, however, through research examining whether these proposed biases appear to be specific to the processing of social information as opposed to more global neuropsychological deficits among individuals with SP.

Neuropsychological evaluation uses paper-and-pencil and computer-based measures that have been previously established to correlate with functioning in particular brain regions. Neuropsychological tests are designed to assess for neurologically-based dysfunction and deficits, whereas the cognitive bias tasks mentioned above are designed to assess for differences that emerge only during the processing of specific types of information. It is essential that research be directed toward ruling out any possible neurologically-based deficits that might otherwise account for current empirical findings suggesting that SP is characterized by biases in the processing of socially-relevant information. Although relatively little research to date has investigated neuropsychological functioning among individuals with SP, there have been a few notable studies [22]–[27]. Unfortunately, these studies have tended to investigate specific domains of neuropsychological performance while excluding other potentially-relevant domains of functioning. Furthermore, a review of the extant literature reveals conflicting findings regarding the performance of SP patients within nearly every domain of neuropsychological functioning.

Several studies have reported decreased performance in the visual-spatial processing domain (i.e., physically and mentally working with visual information) among SP patients as compared to nonpsychiatric controls, as evidenced by scores on Block Design [22], [23] as well as a cube drawing test [24]. However, findings in other domains have been less consistent. For example, significant group differences in the verbal memory domain (i.e., storing verbal information over a relatively long period of time and then retrieving) were reported by both Asmundson et al. [22] and Airaksinen, Larsson, and Forsell [25], although Sachs et al. [26] reported no significant differences for SP patients in this domain.

Similarly, in the domain of executive functioning (i.e., higher-level cognition such as abstraction and reasoning), performance on both the Trail-Making Test (Trail B) and the Wisconsin Card Sorting Test have been examined with mixed results. On the Trail-Making Test (Trail B), several investigations found no significant differences in completion time for SP patients as compared to controls [22], [25], [27]. Conversely, Cohen et al. [23] reported significantly longer completion times on this test for SP patients, suggesting that these individuals experienced greater difficulty in rapid cognitive set-shifting when compared to controls. On the Wisconsin Card Sorting Test, Sachs et al. [26], as well as Graver and White [27], reported no significant differences for SP patients under baseline conditions. SP patients in one study did exhibit declining performance on the Wisconsin Card Sorting Test when performing the task under a stress-induction condition, however [27].

Mixed findings have also been present in the attention domain (i.e., maintaining mental focus). Sachs et al. [26] reported significantly decreased accuracy on the Digit Cancellation Test for SP patients compared to controls, and Asmundson et al. [22] reported a non-significant trend toward reduced accuracy on a similar test of visual scanning and cue recognition. Conversely, both Cohen et al. [23] and Graver and White [27] found no significant differences in performance on the Digit Span Forward subtest.

Currently, it appears that only one published study has examined the visual working memory domain (i.e., the ability briefly retain and mentally manipulate nonverbal stimuli) among individuals with SP. Graver and White [27] reported no significant group differences on Spatial Span during baseline; when exposed to a social anxiety-provoking situation, however, the SP group did show a decrement in performance on this task. Finally, the domain of verbal working memory (e.g., the ability to briefly retain and mentally manipulate verbal information) does not appear to have been adequately assessed by any previous study examining a SP sample.

These inconsistent results across studies are difficult to interpret and may be the result of different samples, diagnostic procedures, measures, or a combination of these variables, among many others. Thus, the few reports of neuropsychological deficits in SP may represent spurious findings from other confounds, and there remains a need for research that examines whether a specific neuropsychological profile is associated with SP. The present research aimed to refine our understanding of potential underlying processes in SP through administration of a comprehensive neuropsychological test battery to a single sample of individuals meeting DSM-IV diagnostic criteria for generalized social phobia.

## Method

### Objective

It was hypothesized that the social phobia group would show a statistically significant reduction in performance, compared to nonpsychiatric controls, in the domains of verbal learning and visual-spatial processing. This is based on the few areas of overlap and potential agreement in the extant literature, which suggests a greater probability of true differences in performance within these particular cognitive domains. It was reasoned that these deficits would be consistent with the clinical symptoms of poor retention for verbal information presented in social situations as well as avoidance of eye contact and preferential visual attention to social threat cues. These symptoms could in turn be related to downstream cognitive processes that serve to maintain the disorder, including biases toward negative evaluation of social feedback cues and decreased confidence in one's own social abilities. For example, decreased verbal memory and learning skills could hinder an individual's ability to retain and recall instances of positive social interactions and verbal feedback, thereby limiting the ability to generate counterexamples to challenge negative thoughts and beliefs regarding his or her social performance. Similarly, decreased visual-spatial processing abilities may prevent an individual from accurately perceiving complex visual cues related to social feedback such as facial expression and body language, which could lead to misinterpretation of others' reactions through a failure to observe and integrate certain salient visual features as part of the total visual scene (e.g., neglecting the emotion conveyed by one's eyes in the context of the larger facial expression).

### Participants

This study recruited 50 participants from the local community: 25 individuals meeting criteria for generalized social phobia (SP) and 25 participants serving as nonpsychiatric controls (NC). Participants were recruited through use of advertisements in newspapers and websites, word of mouth from previous participants, and posted flyers in the community. The advertisements included information about a cash stipend that all participants received in return for participation. Some of the advertisements targeted individuals who were likely to have social phobia, while others targeted nonpsychiatric control participants. We obtained verbal informed consent and conducted a brief phone screen on all individuals who responded to our advertisements. This served to screen out individuals who did not seem appropriate for the diagnostic categories, as well as individuals reporting a history of neurological illness, traumatic brain injury, or other self-reported psychiatric illness or treatment. All participants were between the ages of 18 and 65, and there were no restrictions based on gender, race, or ethnicity. We did, however, attempt to match the demographics of the NC group to those of the SP group (see Table 1). Although an IQ estimate was not obtained for the participants in this study, the two groups would not be expected to differ in IQ based upon their similar levels of education (see Table 1) as well as their similar scores on tests of memory and learning (see Table 2). Participants were excluded from the study if they met diagnostic criteria for psychiatric illnesses other than social phobia (for the SP group), with an allowance for specific phobia in both groups. Other exclusionary criteria for both groups included: (1) a history of significant head injury, neurological illness, or systematic medical diseases that may affect neurocognitive functioning; (2) being currently prescribed certain classes of medication that have a strong potential to decrease cognitive performance (i.e., benzodiazepines, tranquilizers, antipsychotics, or narcotic pain medications); and (3) reporting significant alcohol consumption or any other substance use within the past 48 hours. During the course of recruitment, six individuals were excluded for not meeting diagnostic criteria for SP. In the current sample, two participants in the SP group and one participant in the NC group met criteria for a specific phobia. None of the participants in either group endorsed being prescribed psychotropic medication of any kind, and all participants denied both current and past psychological treatment for social phobia or specific phobia.

**Table 1:** Demographic and Clinical Characteristics.

Measure	Social Phobia Group (*n* = 25)	Nonpsychiatric Control Group (*n* = 25)
Gender (male)^‡^	52%	52%
Age	38.04 (12.85)	38.60 (12.10)
Years of Education	14.40 (1.73)	14.56 (1.71)
Race: Caucasian^‡^	68%	68%
Race: Hispanic/Latino^‡^	16%	8%
Race: Black/African American^‡^	8%	12%
Race: Asian^‡^	4%	12%
Race: Multiracial/Other^‡^	4%	0%
ADIS-IV: CSR	4.56 (0.65)**; range = 4–6	0.20 (0.50)**; range = 0–2
SPAI-23: Social Phobia	63.60 (8.54)**; range = 37–78	30.56 (12.03)**; range = 16–55
SPAI-23: Agoraphobia	19.56 (5.72)**; range = 7–30	10.20 (5.24)**; range = 7–25
SPAI-23: Difference Score	44.04 (8.64)**; range = 30–70	20.36 (10.37)**; range = 9–46
STAI: State	49.76 (8.96)**; range = 26–64	25.52 (5.64)**; range = 20–45
STAI: Trait	57.28 (11.13)**; range = 28–72	31.44 (8.35)**; range = 20–56

** *p*<0.001.

Values represent means and standard deviations for all variables except for those notated (^‡^ indicates a percentage).

**Table 2:** Group Differences by Cognitive Domain.

Domain	Social Phobia Group *z*-scores [*M*, (*SD*)]	Nonpsychiatric Control Group *z*-scores [*M*, (*SD*)]	*t* value	df	*p* value	Effect Size (*d*)
Verbal Learning	−0.155 (0.733)	0.000 (1.000)	0.626	48	0.535	0.177
Verbal Delayed Memory	0.000 (0.983)	0.000 (1.000)	0.000	48	1.000	<0.001
Visual Immediate Memory	−0.232 (1.052)	0.000 (1.000)	0.798	48	0.429	0.226
Visual Delayed Memory	−0.139 (0.934)	0.000 (1.000)	0.507	48	0.614	0.144
Visual-Spatial Processing	0.084 (1.276)	0.000 (1.000)	0.257	48	0.798	0.073
Verbal Working Memory	−0.198 (1.374)	0.000 (1.000)	0.582	48	0.563	0.165
Visual Working Memory	−0.506 (0.728)	0.000 (1.000)	2.043	48	0.047*	0.579
Executive Functioning	−0.166 (1.163)	0.000 (1.000)	0.542	48	0.590	0.153
Attention	0.205 (0.844)	0.000 (1.000)	0.782	48	0.438	0.222

* *p*<0.05.

### Procedure

After completing informed consent, all participants were administered the Anxiety Disorders Interview Schedule - IV [28] to assess for the presence of specific anxiety disorders as well as mood disorders, somatoform disorders, and substance-related disorders. The Social Phobia and Anxiety Inventory - 23 (SPAI-23) [29] and the State-Trait Anxiety Inventory (STAI) [30] also were administered to further examine the presence and severity of anxiety symptoms. Neuropsychological functioning was then assessed with specific subtests from the Wechsler Adult Intelligence Scale - III (WAIS-III) [31] and Wechsler Memory Scale – III (WMS-III) [32], as well as the Rey Complex Figure Test (RCFT) [33], the Trail-Making Test (TMT) [34], and a computerized Stroop task. Each of the neuropsychological measures fell under one of nine cognitive domains: (1) Verbal Learning, assessed by the ability to immediately recall a list of words across multiple presentations; (2) Verbal Delayed Memory, assessed by the ability to recall a previously-presented list of words after a time delay; (3) Visual Immediate Memory, assessed by the ability to immediately recall the details of a set of visual scenes; (4) Visual Delayed Memory, assessed by the ability to recall the details of a set of previously-presented visual scenes after a time delay; (5) Visual-Spatial Processing, assessed by the ability to construct designs using a set of blocks as well as the ability to reproduce and draw a complex visual stimulus; (6) Verbal Working Memory, assessed by the ability to retain auditory information and mentally manipulate that information into a new order according to an established rule; (7) Visual Working Memory, assessed by the ability to retain the sequence in which nonverbal stimuli are presented and reproduce the sequence both in the same order as well as in the reverse order as the original presentation; (8) Executive Functioning, assessed by the ability to rapidly shift between cognitive sets as well as the ability to inhibit an automatic response when presented with color words printed in an incongruent color of ink; and (9) Attention, assessed by the ability to repeat a sequence of numbers as well as the ability to visually scan and identify letters in alphabetical order. The measures which comprised each domain are summarized in Table 3.

**Table 3:** Measures and Raw Scores by Cognitive Domain.

Domain	Measures	Social Phobia Group Raw Scores [*M*, (*SD*)]	Nonpsychiatric Control Group Raw Scores [*M*, (*SD*)]
Verbal Learning	WMS-III Word Lists I	32.200 (4.916)	33.240 (6.704)
Verbal Delayed Memory	WMS-III Word Lists II	6.960 (2.208)	6.960 (2.245)
Visual Immediate Memory	WMS-III Family Pictures I	34.080 (11.445)	36.600 (10.882)
Visual Delayed Memory	WMS-III Family Pictures II	34.560 (10.771)	36.160 (11.528)
Visual-Spatial Processing	WAIS-III Block Design	38.440 (15.338)	36.840 (13.741)
	RCFT (Copy)	32.220 (7.472)	32.100 (4.958)
Verbal Working Memory	WMS-III Letter-Number Sequencing	10.800 (3.055)	11.240 (2.223)
Visual Working Memory	WMS-III Spatial Span	15.520 (2.535)*	17.280 (3.482)*
Executive Functioning	TMT – Trail B	63.884 (20.713)	57.706 (16.927)
	Stroop Task	182.200 (90.705)	166.800 (86.683)
Attention	WMS-III Digit Span (Forward)	10.480 (2.257)	9.760 (2.107)
	TMT – Trail A	25.924 (7.516)	25.926 (9.641)

* *p*<0.05.

### Ethics

All participants in this study provided written informed consent prior to beginning the research procedures detailed above. At the end of the research session, participants were provided with a debriefing statement that discussed the purpose of the study. All participants were also provided with a list of treatment referral sources in the event that they wished to seek psychological services. This study was approved by the University of Central Florida Institutional Review Board.

## Results

### Clinical Interview Data

An estimate of diagnosis accuracy was obtained using a procedure modeled after Turner, Beidel, Long, and Greenhouse [35]. All ADIS-IV interviews were recorded as digital audio files and stripped of all personally-identifying data. Thirteen of these files were randomly selected to be evaluated by an independent researcher not associated with the present study, who was blind to diagnosis. In each of these cases the independent evaluator confirmed all final diagnoses and subsequent assignment to the SP or NC group, resulting in an estimated reliability coefficient of *κ* = 1. As expected, individuals in the SP group received significantly higher clinical severity ratings (CSRs) from the ADIS-IV in regard to symptoms of social anxiety as compared to the control group, *t*(48) = 26.57, *p*<0.001. Means, standard deviations, and ranges for each group on these measures are reported in Table 1.

### Self-Report Questionnaires

As expected, individuals in the SP group reported significantly higher levels of anxiety than controls on the SPAI-23 Social Phobia subscale, *t*(48) = 11.199, *p*<0.001, as well as the SPAI-23 Agoraphobia subscale, *t*(48) = 6.037, *p*<0.001. The SPAI-23 Difference Score (Social Phobia subscale score minus Agoraphobia subscale score) was also significantly higher for the SP group, *t*(48) = 8.772, *p*<0.001. Similarly, individuals in the SP group received significantly higher STAI-State scores, *t*(48) = 11.446, *p*<0.01, and STAI-Trait scores, *t*(48) = 9.289, *p*<0.001, as compared to individuals in the NC group. Means, standard deviations, and ranges for each group on these measures are reported in Table 1.

### Cognitive Tasks

All raw test scores from the cognitive tasks were transformed into *z*-scores using the means and standard deviation values from the NC group. For cognitive domains that contained more than one test score (see Table 3), the respective *z*-scores from the individual tests were averaged to create a z-score for each domain (see Table 2). The *z*-scores served as the dependent variable in a mixed two-factor ANOVA, with group serving as the between-subjects variable and cognitive domain serving as the within-subjects factor. This analysis did not reveal a significant group by cognitive domain interaction, *F*(8, 384) = 0.759, *p* = 0.640, η^2^ = 0.016, nor a significant main effect of group, *F*(1, 48) = 0.445, *p* = 0.508, *η^2^* = 0.009.

As this appeared to be the first study to examine a broad range of neuropsychological domains in a single sample of individuals with social phobia, exploratory univariate analyses were performed to examine group differences within each of the nine neuropsychological domains (see Table 2). Although significant group differences initially emerged within the Visual Working Memory domain such that the SP group demonstrated decreased accuracy relative to the NC group, this finding did not survive a Bonferroni correction for the multiple comparisons (*p*<0.01 for the nine domains), suggesting that this may be a spurious group difference. Results did not indicate significant group differences on any other cognitive domain score.

In light of previous research suggesting decreased neuropsychological test performance among individuals with SP only under conditions of increased stress [27], Pearson correlations were conducted within the SP group to examine whether level of state anxiety (i.e., the STAI-State raw score) was related to performance on any of the nine neuropsychological domains (using the z-scores). No significant correlations emerged between STAI-State score and any of the nine cognitive domain scores for the SP group, however (all *p*'s>.25).

## Discussion

There is a relative paucity of published research examining neuropsychological functioning in SP, and the few studies that do exist have focused on isolated neuropsychological domains and have generally reported mixed findings. Interpretation of these previous findings has been further complicated by methodological inconsistencies across studies. In an effort to clarify whether a distinct neuropsychological profile for SP exists, the current study administered a comprehensive neuropsychological test battery to a single sample of individuals meeting DSM-IV diagnostic criteria for generalized social phobia (SP) as well as a sample of nonpsychiatric controls (NC). Based on the limited literature regarding the neuropsychological functioning of social phobia patients, we hypothesized that the SP group would show a statistically significant reduction in performance, compared to the NC group, in the domains of Verbal Learning and Visual-Spatial Processing. Results obtained from the current sample, however, failed to support both of these hypotheses.

Specifically, a mixed ANOVA did not reveal a significant group by cognitive domain interaction, nor did it reveal a significant main effect of group. Notably, the effect sizes for the main effect of group and the group by domain interaction were very small (η^2^<0.02 in each case), suggesting that it is unlikely that these factors would be statistically significant in a larger sample. Furthermore, no significant group differences were apparent between the SP and NC groups within each of the specific neuropsychological domains.

Despite previous evidence of decreased neuropsychological functioning among SP patients when under conditions of increased stress [27], no significant correlation between level of state anxiety and neuropsychological performance was found among individuals in the SP group (all *p*'s>.25) or the control group (all *p*'s>.09). This lack of relationship in the control group is consistent with some research in nonpsychiatric adults, e.g., [36], but inconsistent with other studies that have reported a negative relationship between state anxiety and neuropsychological performance in particular domains, e.g., [37], [38], [39]. We did find a suggestion of a negative relationship between state anxiety and performance on the neuropsychological domain of Visual Immediate Memory in the control group, which represented a small-to-medium effect size, but this was not statistically significant, *r*(25) = −.34, *p* = .10. It is possible that we would have found significant relationships between state anxiety and neuropsychological performance in particular domains with a larger sample size.

The pattern of results in the present study appears to be consistent with current theoretical models of SP, which assert that cognitive biases should only be observed during the processing of socially-relevant information [1], [2]. The current neuropsychological battery was designed to examine potential neurological deficits, and the stimuli utilized by these traditional neuropsychological measures were relatively free of any social context. Therefore, our lack of significant differences in neuropsychological performance between the SP and NC groups would be expected when considered through the framework of the current theoretical models of SP. Taken together with the growing body of literature supporting specific biases in attention, interpretation, and memory for socially-relevant information among individuals with SP, the present findings may serve as further support for theoretical assertions that cognitive biases are specific to the processing of social (versus non-social) information and do not represent underlying neurological deficits. Indeed, a similar conclusion was reached by O'Toole and Pedersen [40] after conducting a recent review of the literature on neuropsychological performance among adults diagnosed with SP.

### Limitations

The present research is not without limitations. First, the community sample assessed in the current study may have represented a set of individuals with less severe symptomatology and higher overall functioning than is typically seen in clinical settings. For example, the ADIS-IV clinical severity ratings (CSRs) in the SP group ranged from four to six (see Table 1), despite the fact that the CSR scale extends to a rating of eight and that a rating four is generally considered the minimum CSR for those meeting full diagnostic criteria. Moreover, the SPAI-23 Difference score in the SP group (see Table 1) reflected both a lower mean and a narrower range when compared to the original SPAI Difference scores of the clinical sample in the normative group for that measure (*M* = 95.77, *SD* = 32.55, range = 15–160) [29]. It should be noted, however, that all participants in the SP group did meet full diagnostic criteria for the disorder based on the ADIS-IV. The current findings are therefore likely to be representative of SP patients, although it is less clear the extent to which these findings can be generalized to those experiencing more severe forms of the disorder. The current study was also limited by the inclusion of only a single task representing the majority of the cognitive domains, with the exception of the Visual-Spatial Processing, Executive Functioning, and Attention domains. While the measures used in the current study are well-validated and commonly used to assess these various areas of neurocognitive functioning, it is possible that other neuropsychological tests may be more sensitive in detecting differences in cognitive functioning among individuals with SP. Lastly, although the results of the current study do appear to be consistent with current theoretical models as well as the extant literature on information-processing biases in SP, there is always the possibility that null findings are due to an unknown source of error and any interpretation of these results should be considered in light of this limitation.

Future research would likely benefit from a more in-depth investigation of how individuals with SP process both social and non-social information. The results of the current study, as well as previous findings in the literature, seem to indicate that the discrepancy between processing these two types of information is where cognitive biases emerge among individuals with SP. If the nature of these cognitive biases can be further clarified, then this new knowledge can be applied to further refine theoretical models of SP and ultimately enhance current treatment approaches for individuals with SP.
